# Improving Resident Doctors’ Consideration of Spirituality/Religious Beliefs in Clinical Assessments

**DOI:** 10.1192/bjo.2025.10186

**Published:** 2025-06-20

**Authors:** Safia Zaffarullah, Tom Robson, Neelima Reddi

**Affiliations:** Surrey and Borders Partnership NHS Foundation Trust, Surrey, United Kingdom

## Abstract

**Aims:** The spiritual dimension to an individual’s presentation is rarely considered in psychiatric assessments despite the RCPsych’s position statement on spirituality/religion (S/R) and a growing body of evidence. A 2024 SABP trust survey of Resident Doctors indicated a significant disconnect between trainees perceived importance of S/R clinically and actual practice. An audit was thus completed with the following **Aims:** gather baseline data exploring the frequency with which Resident Doctors are recording spirituality in inpatient admissions clerking; provide educational intervention to Resident Doctors tailored to training needs highlighted in survey; following intervention, re-audit to ascertain whether there have been any improvements.

**Methods:** The baseline audit was a retrospective review of admissions clerking recorded for all new admissions over February–March 2024 to 3 inpatient wards at SABP Trust. Each record was manually searched for key words, including spiritual*, religio*, spiritual or religious faith. Anonymised findings were recorded onto an Excel spreadsheet on a secure trust network.

Interventions were then carried out in August and October 2024. Following this, the same wards were re-audited between mid-October and mid-December 2024 using baseline audit criteria.

**Results:** Baseline audit: 1 of 53 new admission records mentioned patient’s S/R beliefs – this was the patient’s own description of their pre-morbid personality. Intervention 1: Segment dedicated to encouraging “spiritual history” taking in the “History Taking” presentation of the SABP Resident Doctors induction programme in August 2024. Intervention 2: An external speaker (Consultant and executive member of RCPsych’s Spirituality SIG) was invited to provide an interactive session at the trust-wide academic programme for Doctors in October 2024, addressing key survey findings. Re-audit: 2 of 45 admission records had mention of patient’s S/R beliefs. One was the patient describing own religiosity in context of religious delusions. In the other, the Doctor had created a “Spirituality” subheading in their clerking record to record patient’s beliefs.

**Conclusion:** This audit indicates that Resident Doctors are still not routinely including spirituality/religious beliefs in clinical assessments despite tailored interventions. Ongoing barriers include reluctance to consider the role of spirituality within mental health care; this being rooted in pervasive cultural stigmas that cannot be fully addressed through one-off interventions.

A cultural shift may only manifest if spiritual history enquiry is recognised as a deserved and crucial component of psychiatric history taking. We thus call for medical educators to consider a “Spiritual History” subheading in their Psychiatry history proforma to promote a collective shift toward more holistic mental healthcare.

